# Loss of clear cell characteristics in aggressive clear cell odontogenic carcinoma: a case report

**DOI:** 10.1186/s13000-024-01530-0

**Published:** 2024-08-13

**Authors:** Yanan Sun, Bo Li, Yaying Hu, Fu Chen, Junchen Pan, Yi Zhou, Jiali Zhang

**Affiliations:** 1 State Key Laboratory of Oral & Maxillofacial Reconstruction and Regeneration, Key Laboratory of Oral Biomedicine Ministry of Education, Hubei Key Laboratory of Stomatology, School & Hospital of Stomatology, Wuhan University, Luoyu Road 237, Wuhan, 430079 PR China; 2 Oral Histopathology Department, School and Hospital of Stomatology, Wuhan University, Wuhan, 430079 China; 3 Oral Radiology Department, School and Hospital of Stomatology, Wuhan University, Wuhan, 430079 China

**Keywords:** Clear cell odontogenic carcinoma, Squamous differentiation, Recurrent, *EWSR1:ATF1* gene fusion

## Abstract

**Background:**

Clear cell odontogenic carcinoma (CCOC) is an odontogenic carcinoma characterized by sheets and islands of vacuolated and clear cells. The diagnosis of atypical CCOC can pose a challenge when tumor cells deviate from their characteristic clear morphology, even with the aid of genetic profiling for CCOC identification.

**Case presentation:**

In this manuscript, we detailed the inaugural instance of a recurrently recurring clear cell odontogenic carcinoma (CCOC) with pronounced squamous differentiation in a 64-year-old male. The primary tumor in this individual initially displayed a biphasic clear cell phenotype. However, subsequent to the third recurrence, the clear tumor cells were entirely supplanted by epidermoid cells characterized by eosinophilic cytoplasm, vesicular chromatin, and prominent nucleoli. Notable aggressive attributes such as necrosis, conspicuous cytological malignancy, perineural dissemination, and vascular invasion were noted. Additionally, the tumor progressed to manifest lung metastases. The tumor cells exhibited positive immunoreactivity for AE1/AE3, KRT19, Pan-CK, EMA, P40, P63, CK34βE12, and P53, while they tested negative for CK35βH11, KRT7, S-100, and neuroendocrine markers. The Ki-67 proliferation index was calculated at an average of 15%. Furthermore, FISH analysis unveiled the presence of the *EWSR1::ATF1* gene fusion.

**Conclusions:**

This case illustrated a rare and aggressive case of CCOC characterized by significant squamous differentiation upon recurrence of the tumor.

**Supplementary Information:**

The online version contains supplementary material available at 10.1186/s13000-024-01530-0.

## Background

Clear cell odontogenic carcinoma (CCOC) is a rare type of odontogenic carcinoma, characterized by sheets of islands of vacuolated and clear cells. According to 2005 and 2017 WHO Classification of Head and Neck Tumors, CCOC can be classified into three subtypes: monophasic, biphasic, and ameloblastoma-like types [1, 2]. In the updated WHO classification of Head and Neck Tumor in 2022, the description of the three histological subtypes of CCOC was removed. In aggressive CCOC, necrosis, conspicuous cytological malignancy, and perineural infiltration can be observed [1]. Despite the generally indolent behavior of some CCOC cases, it is important to note that approximately 20% of reported cases have been found to metastasize, and 42% have experienced recurrence. Molecular studies have indicated that approximately 80% of CCOC cases harbor EWSR1 rearrangements [2]. This case describes an aggressive CCOC with *EWSR1::ATF1* gene fusion, which lost its clear cell characteristics and underwent prominent squamous differentiation after repeated recurrence.

## Case presentation

### Medical and histopathology history in 2012

**Fig. 1:**
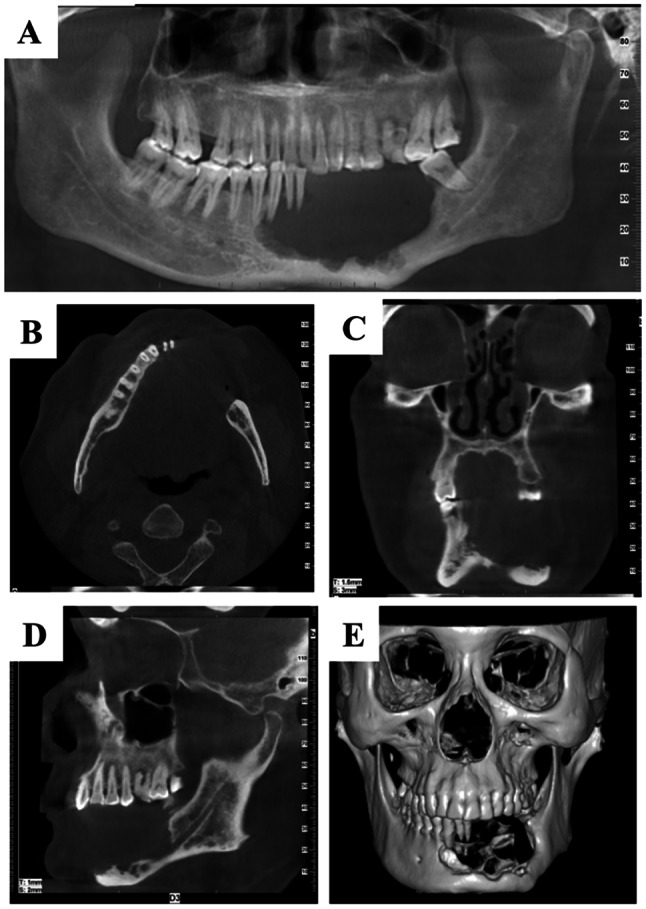
Radiology examination in 2012. (**A**) Panoramic radiographs. (**B**) Transverse plane. (**C**) Coronal plane. (**D**) Sagittal plane. (**E**) 3D-reformatted CT scan

In 2012, a 56-year-old male reported a sensation of looseness in his lower anterior teeth, ultimately resulting in the sudden loss of one of the lower anterior teeth. A cone-beam computed tomography (CBCT) examination revealed a bone defect spanning from teeth 38 to 44, a localized depression near the upper border of the mandible, and rough edges in the affected area (Fig. 1). Subsequently, the patient underwent a wide resection of the mandible from teeth 38 to 44, followed by the reconstruction of the defect using a peroneal myocutaneous flap.

**Fig. 2:**
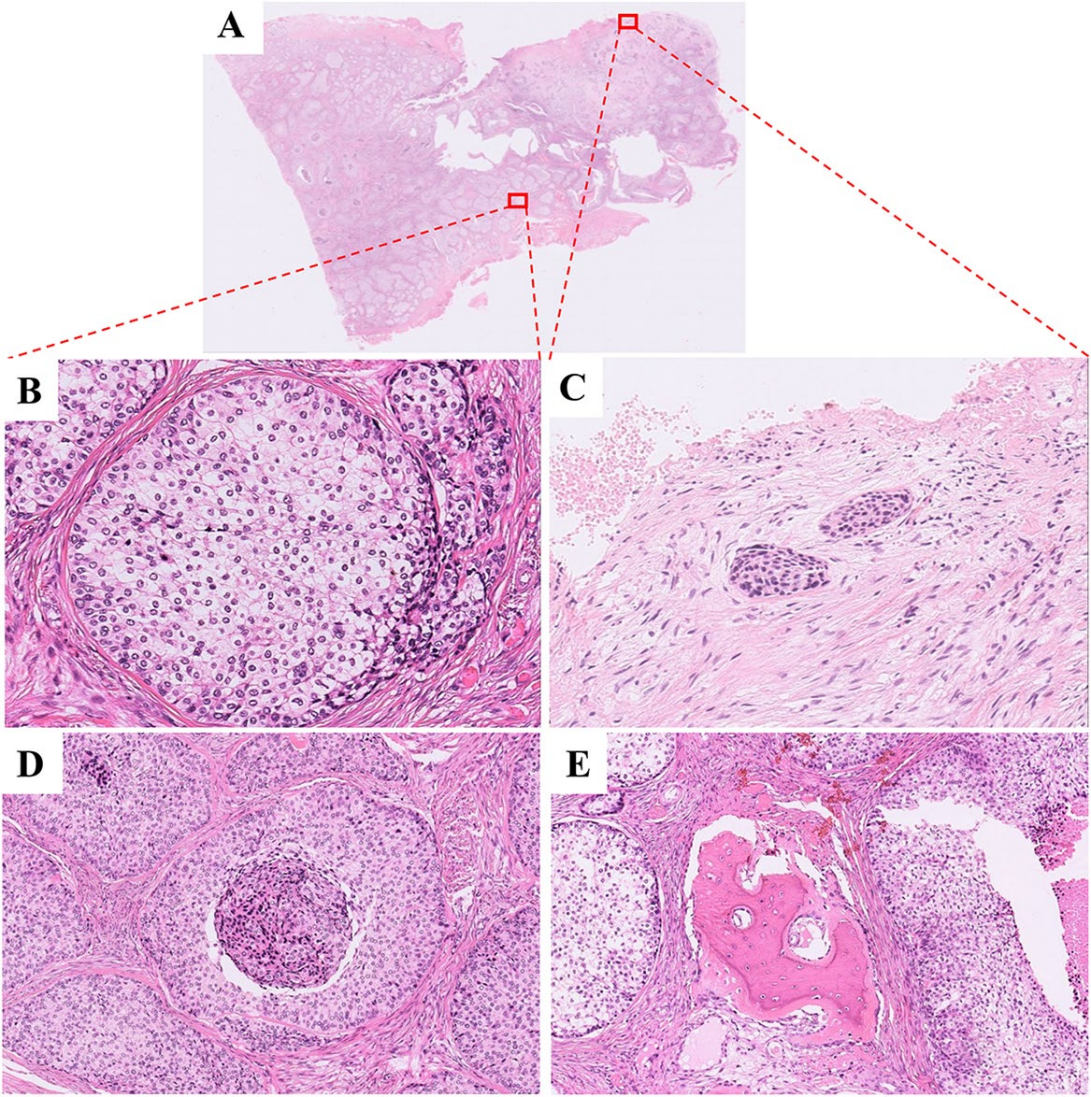
Histopathology of tumor sample in 2012. (**A**) Tumor was composed of varying sized epithelial nests dominated by clear cells, with dense collagen fibers forming fibrous septa (H&E; ×3). (**B**) The epithelial nests arranged in a biphasic pattern, consisting of clear cells and dark, unvacuolated basaloid (H&E; ×200). (**C**) Epidermoid cells with eosinophilic cytoplasm at the edge of the tumor (H&E; ×200). (**D**) Necrosis was observed within the epithelial nest of tumor (H&E; ×100). (**E**) The tumor cells invading bone tissue (H&E; ×100)

The histological examination revealed that the tumor consisted of epithelial nests of varying sizes arranged in a biphasic pattern. These nests comprised predominantly clear cells along with peripheral dark, unvacuolated basaloid cells (Fig. 2A, B). At the lesion’s periphery, a small subset of tumor cells displayed characteristics of epidermoid cells, characterized by eosinophilic cytoplasm. Each nest of epidermoid cells typically contained only a few dozen cells (Fig. 2C). Pathologic mitosis and necrosis were observed within the epithelial nest of tumor (Fig. 2D). The tumor cells were observed to be invading the bone tissue (Fig. 2E). The tumor cells were found to be positive for AE1/AE3, KRT19, Pan-CK, EMA, P40, and P63, and negative for KRT7, S-100, and P53 (Figure S1A-I). The Ki-67 proliferation index averaged 5% (Figure S1J).

### Medical and histopathology history in 2015

**Fig. 3:**
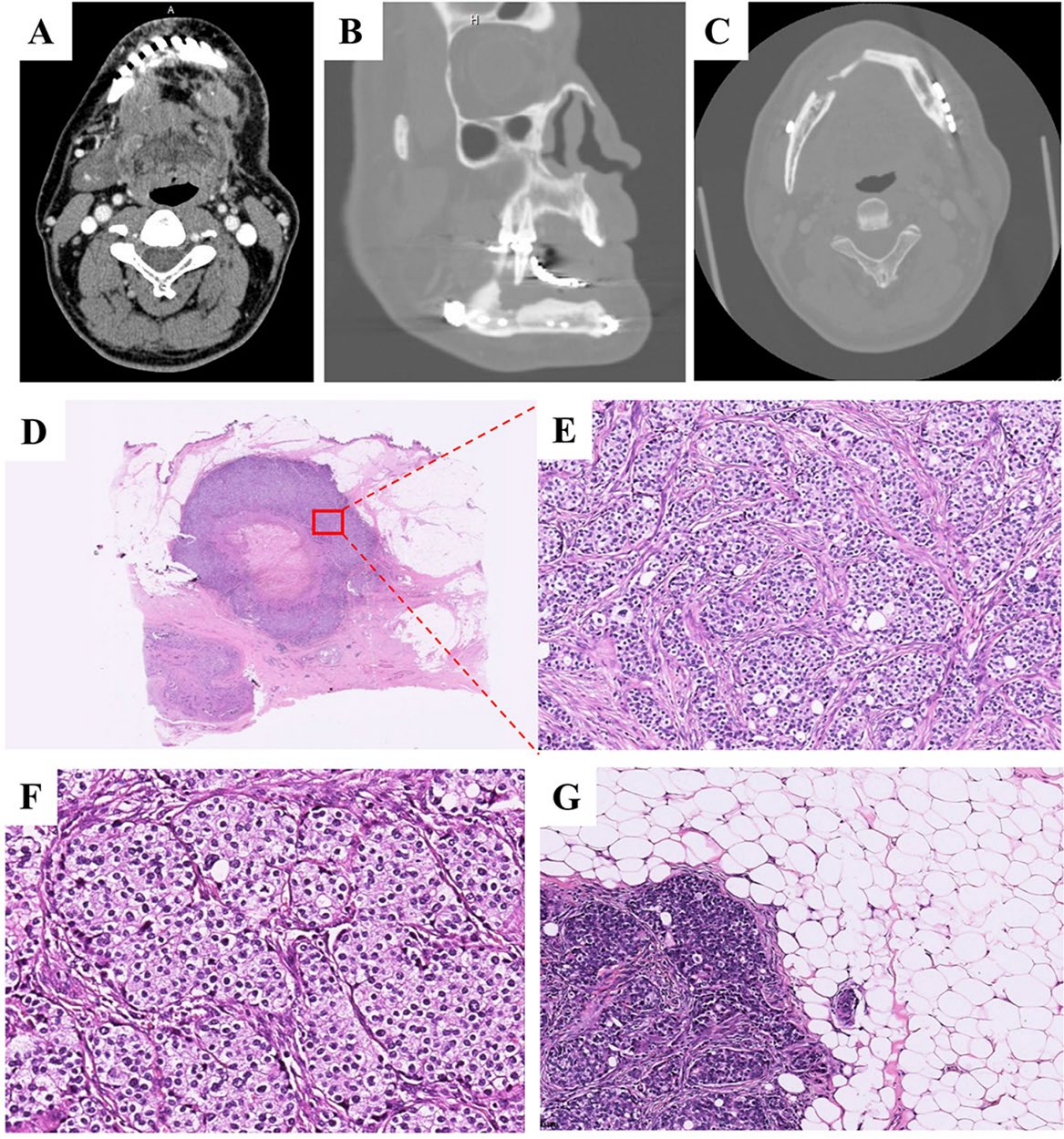
Radiology and histopathology of recurred tumor in 2015. (**A**) An enlarged mass in the submental region. (**B and C**) An ill-defined radiolucent lesion in the right posterior alveolar bone of the mandible (**D, E and F**) Tumor cells are mainly monophasic, with nuclei of different sizes (H&E; D:×3, E:×100; F×200). (**G**) The tumor invaded the surrounding adipose tissue. At the invasion front, tumor cells exhibit an epidermoid morphology (H&E; ×100)

In 2015, a firm mass was palpable in the left submandibular area of the patient, measuring 1.5 × 1.5 × 1.2 cm, exhibiting close adherence to the mandible. No palpable lymph nodes were evident in the neck during this period. CBCT scan showed an enlarged mass in the submental region (Fig. 3A). Furthermore, an ill-defined radiolucent lesion was observed in the right posterior alveolar bone of the mandible. (Fig. 3B-C). The patient underwent surgical resection of the mass, extraction of 45, and resection of the surrounding bone of 45 under general anesthesia.

**Fig. 4:**
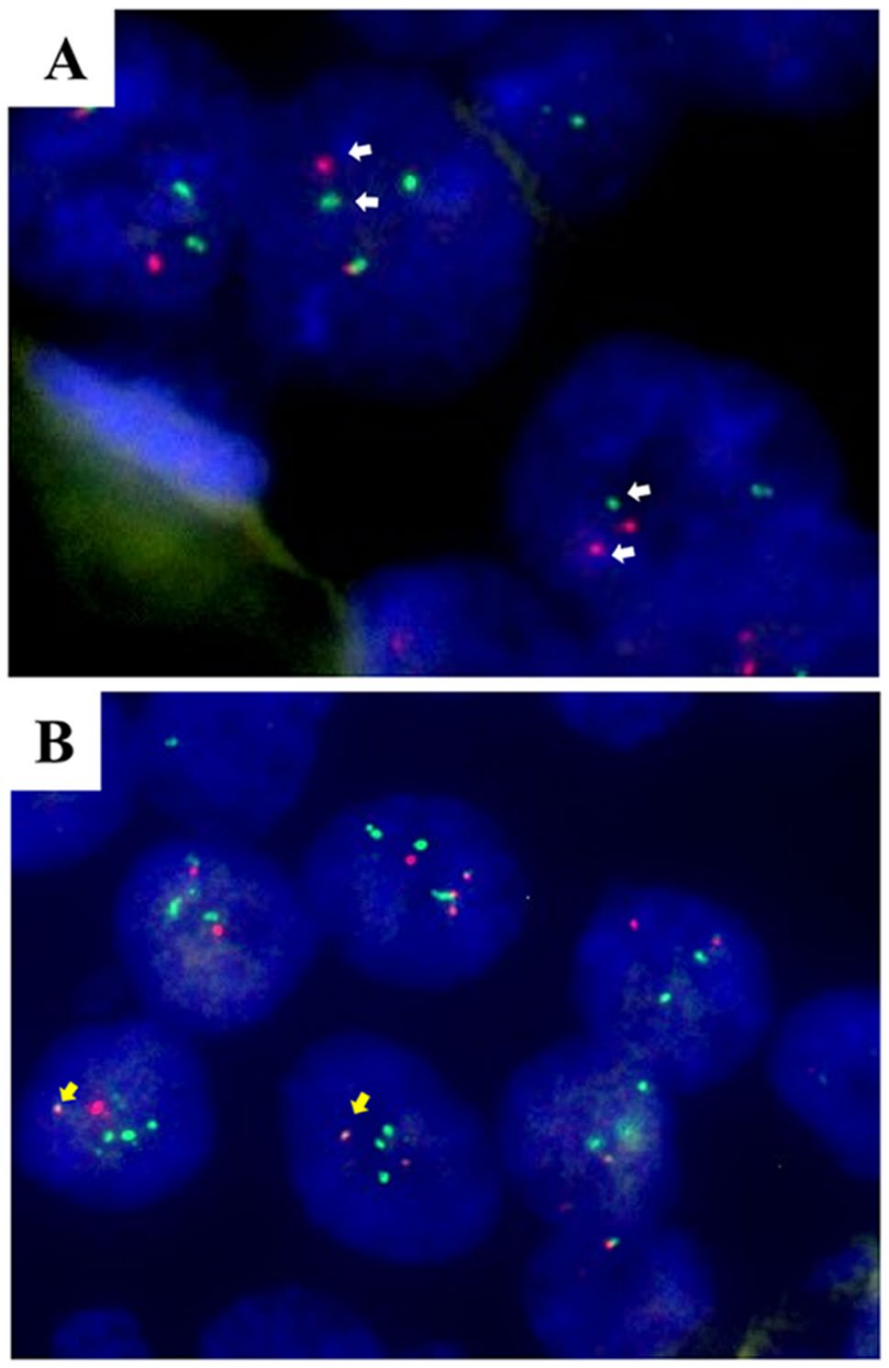
FISH analysis of lesion in 2015. (**A**) Rearrangement of the *EWSR1* (97%) and gene fusion of *EWSR1::ATF1* (80%, **B**). (DAPI, ×1000)

Histologically, the tumor primarily presented as a monophasic variant, with nuclei of different sizes (Fig. 3D-F). At the invasive edge of the tumor, tumor cells exhibited an epidermoid morphology (Fig. 3G). The immunohistochemical marker findings were generally in line with those observed in the 2012 sample (Figure S2). Compared to the specimen from 2012, KRT7 and P53 exhibited weak and focal positivity. (Figure S2C and I). The Ki-67 proliferation index displayed a notable rise, averaging 15% (Figure S2J), suggesting heightened proliferative activity among the tumor cells. FISH analysis showed a rearrangement of the *EWSR1* gene (97%, Fig. 4A) and a gene fusion of *EWSR1::ATF1* (80%, Fig. 4B). The mass was diagnosed as recurrent CCOC. Following the surgery, the patient did not undergo radiotherapy or chemotherapy.

### Medical history in 2018

**Fig. 5:**
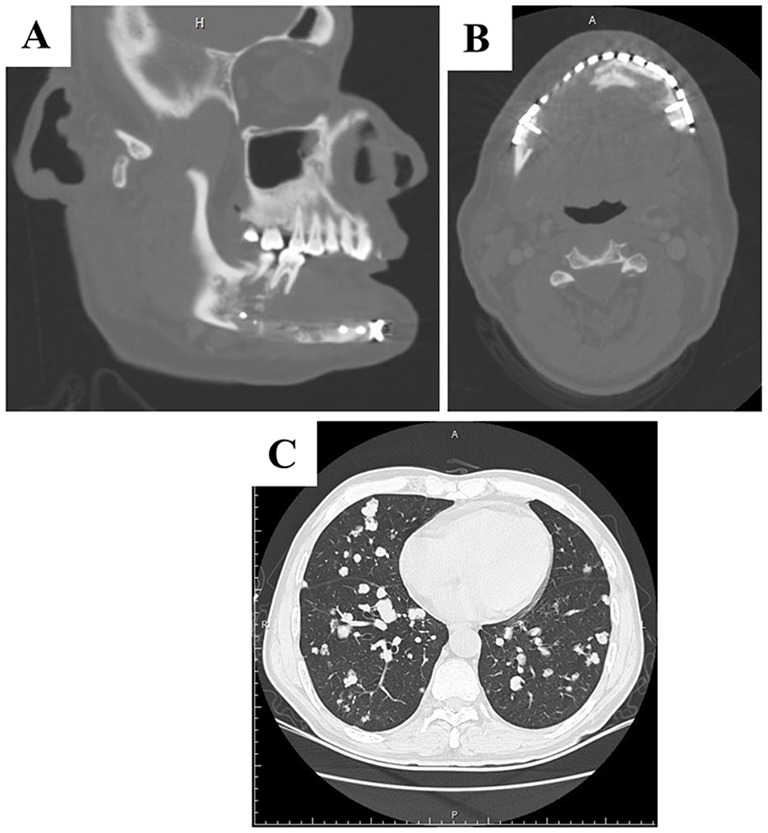
Radiology examination in 2018. (**A and B**) An expanded osteolytic lesion present in both anterior regions the right side of the mandible. (**C**) Typically periphery and circular nodules of varying sizes, scattered in the whole lung

In 2018, the tumor recurred, alongside bilateral lung metastases (Fig. 5). Before the surgical intervention, the patient underwent targeted therapy and chemotherapy. The treatment regimen consisted of Apatinib Mesylate at a dosage of 250 mg once daily and Tegafur/Gimeracil/Oteracil Potassium Capsules at 60 mg twice daily, administered orally. Nevertheless, owing to the occurrence of headaches, the administration of Tegafur/Gimeracil/Oteracil Potassium Capsules was ceased after one week, with only Apatinib Mesylate being continued for a month. Subsequently, the patient underwent an extended mandibulectomy, defect repair involving the transfer of a fibular myocutaneous flap, and submandibular lymph node dissection at another medical facility, where metastasis of the tumor to the submandibular lymph nodes was noted. Post the surgical intervention, the patient did not proceed with radiotherapy or chemotherapy.

**Fig. 6:**
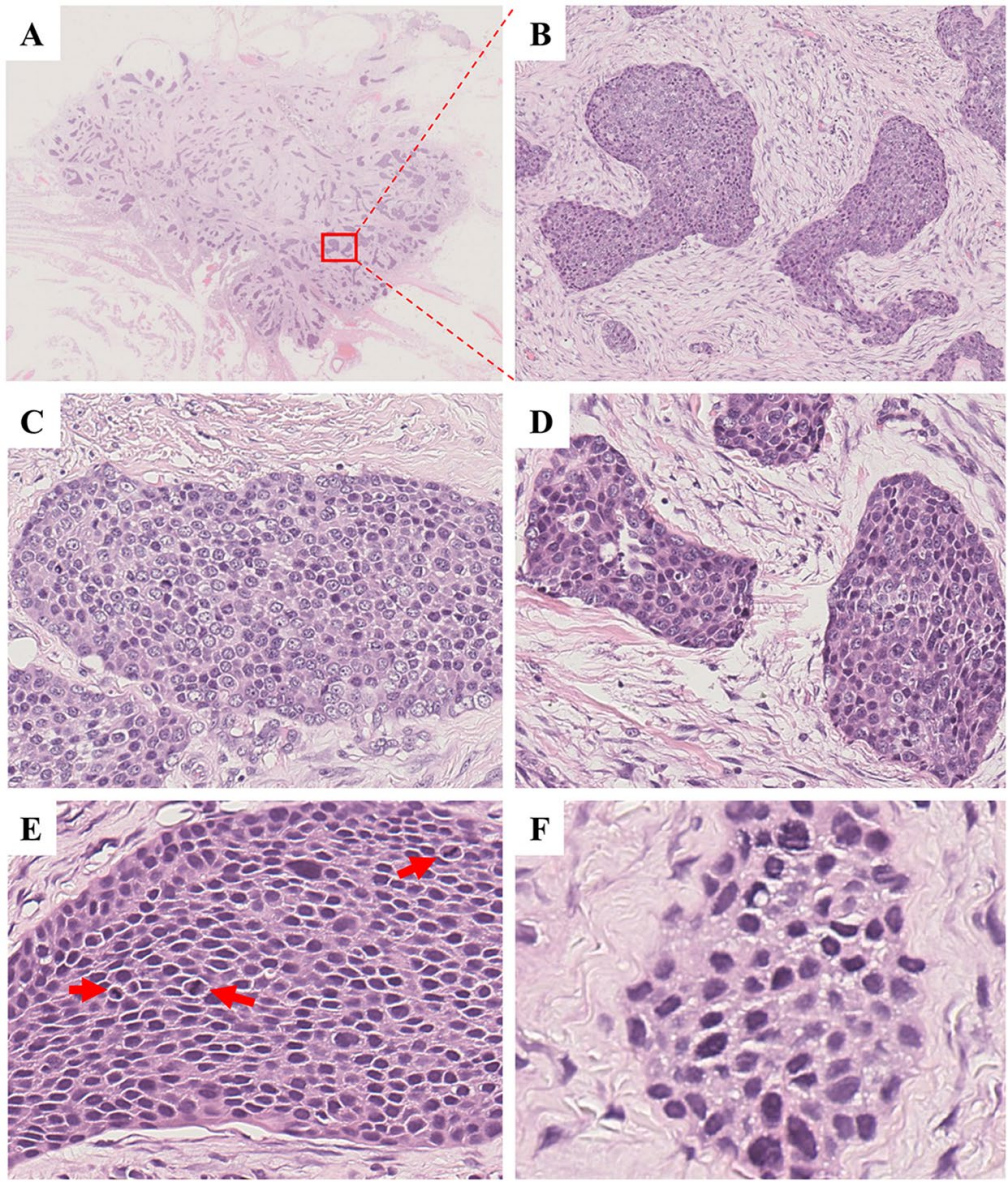
Histopathology of recurred tumor in 2020. (**A**) Low power morphology of CCOC. (H&E, ×5). (**B**) The tumor consists of small and irregular sheets or cords of eosinophilic cells separated by dense collagenous stroma (H&E; ×200). (**C, D**) Tumor cells are relatively uniform in size, nucleus is basophilic. Some of the nucleus are transparent, with glaring nucleolus (H&E; ×300). Abnormal mitosis (H&E; ×300) and (**E**) and the nucleoplasm ratio of tumor cells are increased (H&E; ×400)

### Medical and histopathology history in 2020

In 2020, at the age of 64, the patient presented with a 3.5 × 3 × 1.5 cm mass in the right submandibular region. A CBCT scan conducted at another medical facility unveiled a lesion spanning from 38 to 46, characterized by partial depression along the upper border of the mandible near the chin, delineated by a rugged contour. Subsequently, the patient underwent a right neck dissection, excision of the tumor located in the parapharyngeal space via an external cervical approach, and resection with transplantation of the pediculated fascial flap.

**Fig. 7:**
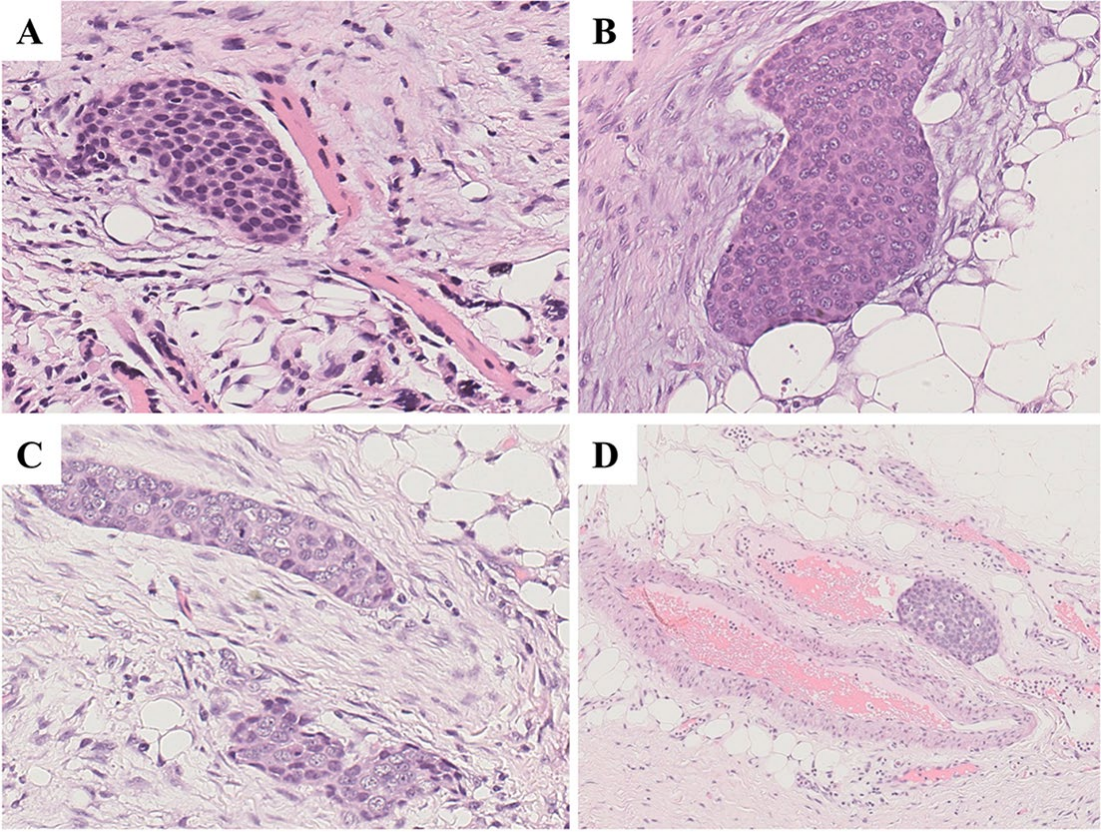
Histopathology of recurred tumor in 2020. The tumor cells invading (**A**) muscle (H&E; ×200), (**B**) adipose tissue (H&E; ×200), (**C**) nerve (H&E; ×200) and (**D**) vessel (H&E; ×100)

Histologically, the recurrent tumor was composed of varying sized epithelial nests of epidermoid cells, with dense collagen fibers forming fibrous septa (Fig. 6A-B). Tumor cells were mildly atypical with eosinophilic cytoplasm, vesicular chromatin, and prominent nucleoli (Fig. 6C-D). At higher magnification, abnormal mitosis was occasionally observed in tumor cells, and the nucleoplasm ratio of tumor cells are increased (Fig. 6E-F). The tumor exhibited aggressive features, including necrosis, destruction of muscle and adipose tissue, perineural spread, and vascular invasion (Fig. 7A-D).

**Fig. 8:**
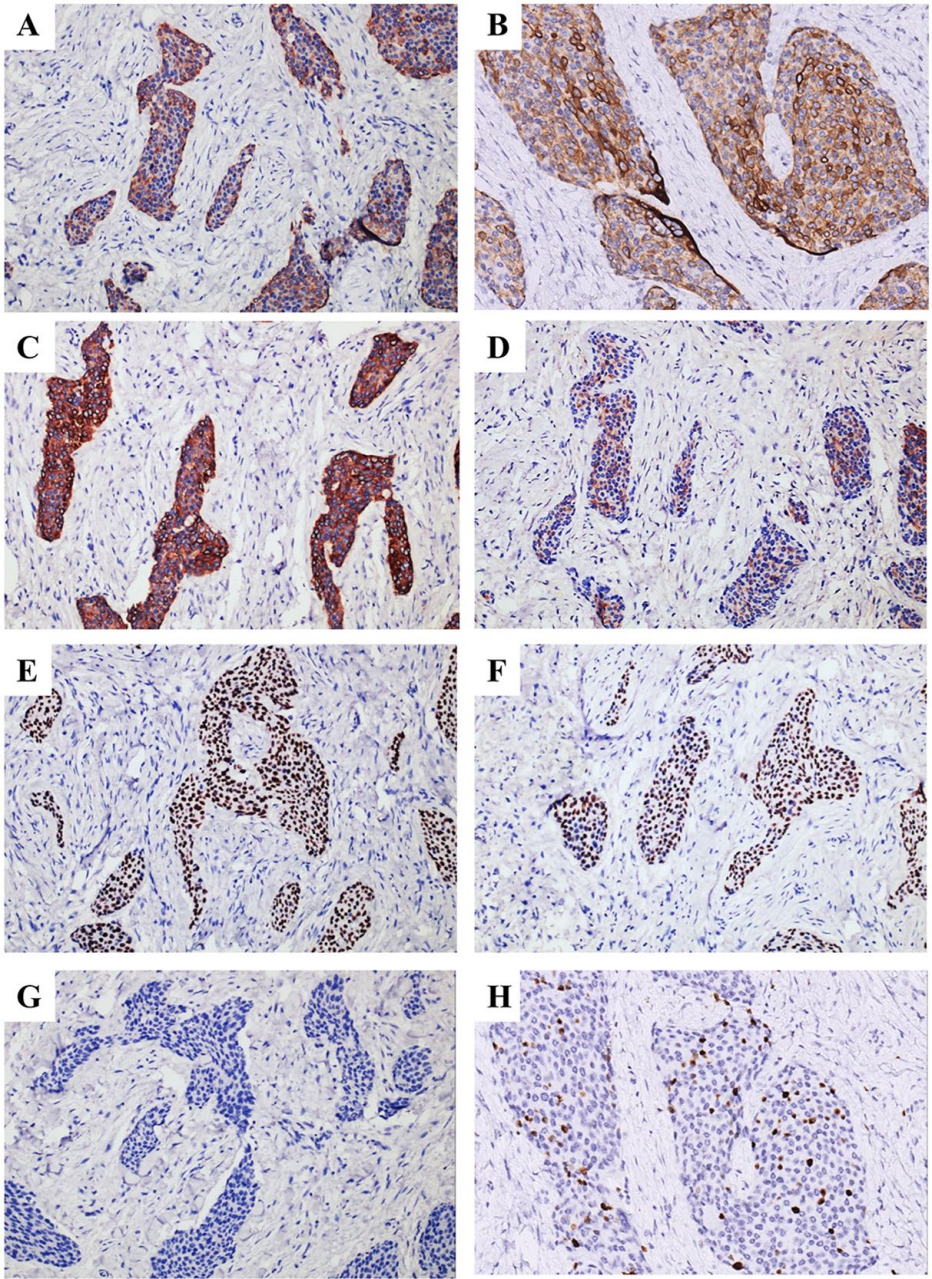
IHC staining of recurred lesion in 2020. The tumor cells were positive for (**A**) AE1/AE3, (**B**) KRT19, (**C**) Pan-CK, (**D**) EMA, (**E**) P40 and (**F**) P63, and negative for (**G**) S-100. (**H**) The Ki-67 proliferation index averaged 15% (IHC, ×200)

**Fig. 9:**
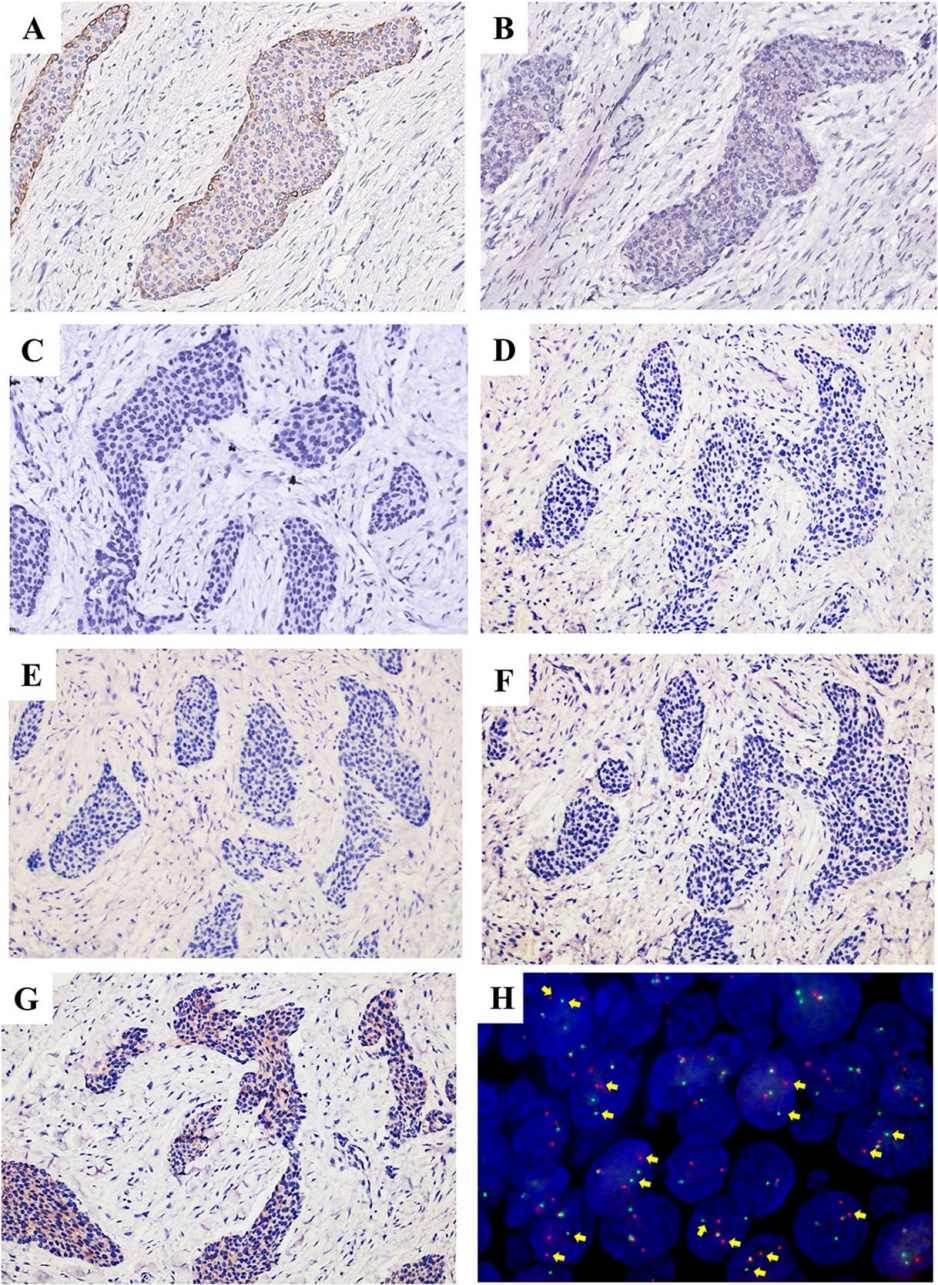
IHC staining and FISH analysis of recurred lesion in 2020. The tumor cells were positive for (**A**) CK34βE12 and negative for (**B**) CK35βH11 and (**C**) KRT7. Neuroendocrine markers, including (**D**) CD56, (**E**) CgA, and (**F**) Syn were negative in tumor cells. Tumor cells were negative for (**G**) CD99. (IHC, ×200). (**H**) EWSR1 dual color break-apart probe hybridized to tumor nuclei. Multiple tumor nuclei are seen with break-apart (yellow arrows) (DAPI, ×1000)

The tumor cells were positive for AE1/AE3, KRT19, Pan-CK, EMA, P40, and P63 (Fig. 8A-F), and negative for S-100 (Fig. 8G), which were consistent with findings in 2012 and 2015 samples. Ki-67 proliferation index averaged 15% (Fig. 8H). Moreover, due to the tumor cells appeared as prominent squamous differentiation, and some regions mimic neuroendocrine differentiation, non-keratinizing squamous cell carcinoma and neuroendocrine carcinoma should be ruled out. The tumor cells were positive for CK34βE12, and negative for CK35βH11 and KRT7 (Fig. 9A-C). Neuroendocrine markers, including CD56, CgA and Syn were negative in tumor cells (Fig. 9D-F). The weak cytoplasmic expression of CD99 rules out the possibility of Ewing’s sarcoma (Fig. 9G), which also harbors *EWSR1* gene rearrangement. FISH was performed to assess the rearrangement of *EWSR1*. Of the 100 tumor nuclei counted for *EWSR1* break-apart probe, 96 nuclei were found to exhibit positive signals (Fig. 9H). The main differential diagnosis and immunohistochemistry panel of this case were listed in Table 1.

**Table 1:** The main differential diagnosis and immunohistochemistry panel of this case

	Epithelium Markers	Neuroendocrine Markers	Other markers	Gene rearrangement
**Poorly differentiated mucoepidermoid carcinoma**	KRT7(+), KRT19(-)	Syn(-), CD56(-), CgA(-)	S-100(-)	*MAML2* rearrangement
**Nonkeratinizing squamous cell carcinoma**	AE1/AE3(+), Pan-CK(+), CK34βE12(+), CK35βH11(-), KRT7(-), EMA(focal+), P63(+), P40(+),	Syn(-), CD56(-), CgA(-)	S-100(-)	Not applicable
**Neuroendocrine carcinoma**	AE1/AE3(+), Pan-CK(+),	Syn(+),CD56(+), CgA(+)		Not applicable
**Ewing sarcoma**	Positive expression in 30% cases	Positive expression in 50% cases	CD99(diffusely and strongly positive in membrane)	*EWSR1* rearrangement
**This case (Recurrent CCOC)**	AE1/AE3(+), Pan-CK(+), CK34βE12(weak+), CK35βH11(-), KRT7(-), KRT19(+), EMA(focal+), P63(+), P40(+),	Syn(-), CD56(-), CgA(-)	CD99 (weakly positive in cytoplasm), S-100(-)	*EWSR1* rearrangement

**Fig. 10:**
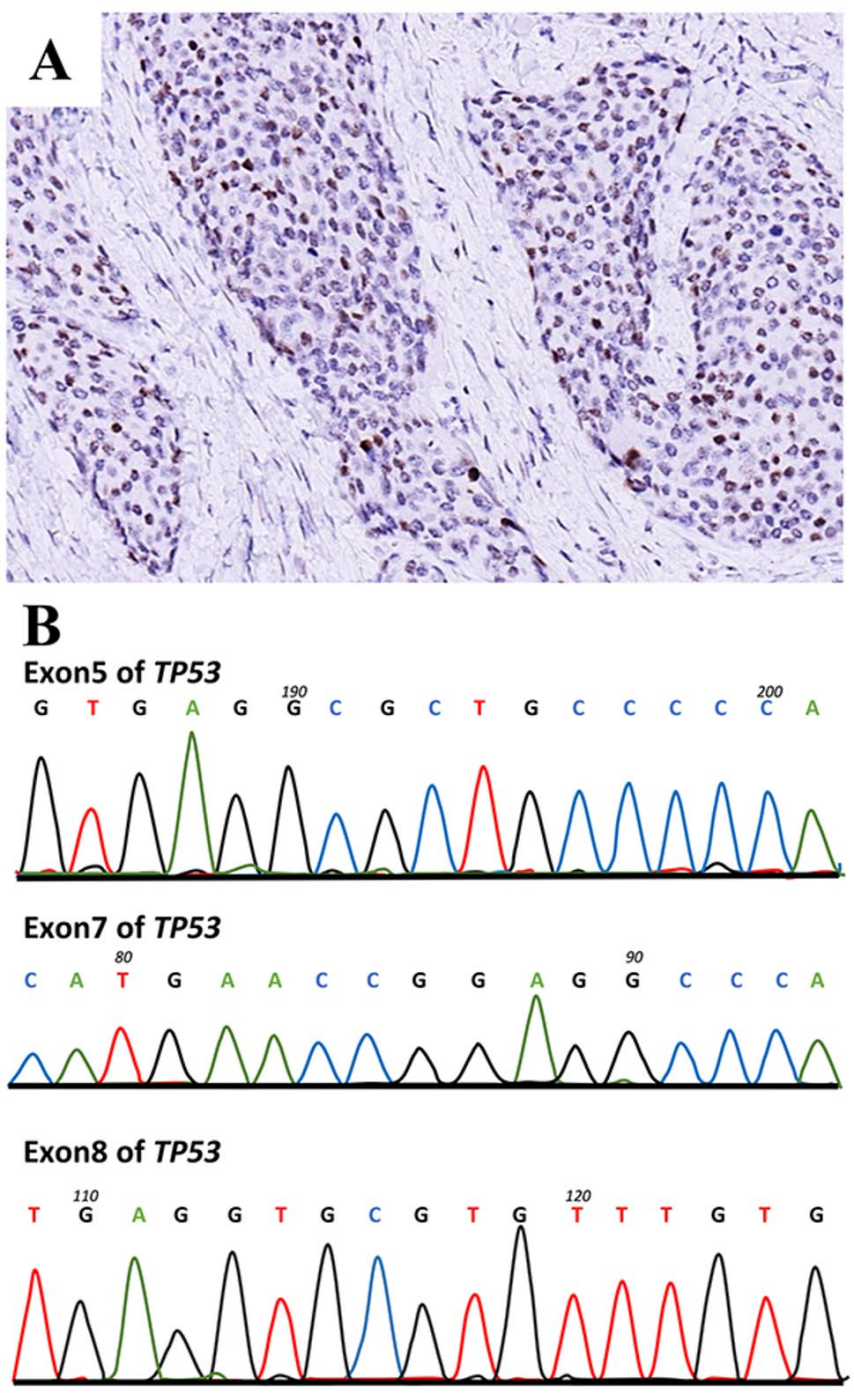
No mutations were detected at the frequently mutated *TP53* locus. (**A**) IHC staining of P53 in recurred tumor in 2020. (**B**) Sanger sequence of exon5, 7 and 8 of TP53

The immunostaining of P53 exhibited a progressive augmentation in tandem with the advancement of the tumor. In the year 2012, the tumor displayed a lack of P53 staining (Figure S1I), while manifesting focal positivity in 2015 (Figure S2I). Notably, by 2018, the tumor cells demonstrated a widespread positivity for P53 (Fig. 10A). In order to ascertain if the aggressive progression was attributed to *TP53* mutations, we conducted Sanger sequencing for exons 5, 7, and 8, which encompass high frequency mutation loci within the *TP53* gene. The results reveal the absence of common *TP53* mutations in any of the tumor samples from 2012, 2015, and 2020 (Fig. 10B).

Unfortunately, the patient passed away due to an accident without receiving any further treatment following the surgical procedure.

## Discussion and conclusions

Clear cell odontogenic carcinoma is a rare odontogenic tumor that first described by Hansen et al. in 1985. According to 2005 and 2017 WHO Head and Neck Tumor Classification, CCOC is categorized into three subtypes histologically: biphasic variant, ameloblastoma-like variant, and monophasic variant. In the biphasic variant, tumor nests were primarily comprised of clear cells, with a few basal-like cells visible in the surrounding layer of the epithelial nest. The cytoplasm of basal-like cells was weakly eosinophilic. In the ameloblastoma-like type, the structure of tumor nests resembles that of an ameloblastoma, with surrounding cell nuclei exhibiting inverted polarity and forming a palisade. The monophasic type consists entirely of clear cells. Up to this point, approximately 131 cases of CCOC have been documented in scholarly works. The histological subcategories of CCOC have been elucidated in 85 publications, encompassing a total of 116 cases. Notably, the biphasic variant predominated with 97 occurrences. Conversely, the ameloblastoma-like variant and monophasic variant were less common, with only 14 and 5 cases reported, respectively. However, in 2022 WHO Head and Neck Tumor Classification, the histological categories of CCOC have been eliminated, indicating histological subtypes may not correlate with the biological behavior and prognosis of the tumor. Other atypical pathological features, including cystic degeneration [3], keratin pearls formation [4] and detinoid deposition [4], have also been observed in a few cases.

Upon reviewing the English literature, CCOC exhibited an invasive growth pattern, with a recurrence rate estimated at 42%. It is noteworthy that only a single published study detailed the histopathology of recurrent CCOC [5]. Omar Breik et al. noted that in recurrent tumors, clear cells were supplanted by clusters, cords, trabeculae, and sheets of neoplastic epithelial cells [5]. An intriguing observation from our investigation in this case is the transition from the initial clear cell phenotype to a prominent squamous differentiation CCOC following multiple recurrences.

In clear cell tumors, the transparency of the cytoplasm, which does not stain in HE, was traditionally attributed to the accumulation of glycogen and deposition of fat [6, 7]. During aggressive progress of the tumor, metabolic alterations can result in heightened glycogen consumption stored within the cytoplasm of tumor cells. This phenomenon may partially elucidate the loss of clear cell characteristics in the tumor cells. The research published in *Nature* in 2013 shed light on the genetic changes associated with clear cell renal cell carcinoma. It also presented evidence of metabolic shifts in aggressive and recurrent clear cell renal cell carcinoma, such as the down-regulation of genes involved in the tricarboxylic acid (TCA) cycle, up-regulation of glutamine transporter genes, and increased levels of acetyl-CoA carboxylase protein. These metabolic alterations are crucial in understanding the progression and behavior of this type of cancer [8]. Although the mechanisms remain unclear, the disappearance of the transparent phenotype of CCOC might relate to the increased consumption of glycogen and aggressive progression.

The diagnostic considerations in this case encompassed neoplasms consist of epidermoid cells, including poorly differentiated mucoepidermoid carcinoma, nonkeratinizing squamous cell carcinoma, and neuroendocrine carcinoma. Histologically, poorly differentiated mucoepidermoid carcinoma exhibited a solid growth pattern with a decreased presence of mucous cells and an increased abundance of epidermoid cells, demonstrating heightened cytologic atypia, necrosis, and perineural invasion. Mucoepidermoid carcinoma typically displayed robust positivity for KRT7 and negativity for KRT19. Additionally, a majority of mucoepidermoid carcinoma harbored rearrangement in the *MAML2* gene. Nonkeratinizing squamous cell carcinoma was characterized by its relative immaturity, minimal to no keratinization, nuclear atypia, numerous mitotic figures, and peripheral palisading of tumor nuclei. The tumor comprised interconnecting squamous sheets that invade the stroma with a broad, pushing border. Immunohistochemically, nonkeratinizing squamous cell carcinoma was positive for high-molecular-weight cytokeratin, p63, and p40. Neuroendocrine carcinoma was composed of cells with hyperchromatic nuclei, indistinct nucleoli, and scant cytoplasm. The presence of numerous mitoses and apoptotic cells is notable. At least one neuroendocrine marker, such as Syn, CgA, or CD56, was typically immunopositive in neuroendocrine carcinomas.

The rearrangement of the *EWSR1* gene was crucial evidence for diagnosing CCOC in this case. *EWSR1* gene rearrangements can be detected in various benign and malignant lesions, including soft tissue and bone entities. In this particular case, *ATF1* was identified as the partner gene involved in the *EWSR1* rearrangement. *ATF1*, *CREB1*, and *CREM* are members of the *CREB* (cAMP response element-binding protein) family and are among the most common partner genes found in *EWSR1* rearrangements. This genetic rearrangement plays a significant role in the pathogenesis and diagnosis of certain tumors, providing important molecular information for accurate classification and management [5, 9, 10]. So far, within all documented instances of CCOC harboring EWSR1 gene rearrangement, the partner genes identified were *ATF1/CREB1/CREM*. In addition to CCOC, the EWSR1-ATF1 gene fusion has been observed in clear cell carcinoma (CCC) of salivary gland, hyalinizing clear cell carcinoma (HCCC) [11], angiomatoid fibrous histiocytoma [12], malignant mesothelioma [13] and atypical central neurocytoma [14]. According to the same molecular alterations, Xuan et al. suggest that it is reasonable to include HCCC as a subtype of CCC [13]. Both CCOC, CCC and HCCC are characterized by the presence of clear cells, leading to arguments suggesting that they are essentially analogous tumors manifesting in distinct anatomical sites. CCOC typically arises in the jaw, while HCCC emerges in the submucosa. Differential diagnosis between these entities relies on supportive evidence from pathological features and tumor localization [15–17].

In this report, we described a novel recurring CCOC with high-grade transformation and disappearance of the transparent phenotype of tumor cells. Because this case is a rare phenotype of CCOC, more cases and a longer follow-up period are necessary to further elucidate its biologic behavior, prognosis, and genetic profile.

### Electronic supplementary material

Below is the link to the electronic supplementary material.

Supplementary Material 1: Fig. 1 IHC staining of tumor in 2012. (A) AE1/AE3, (B)KRT19, (C) KRT7, (D) Pan-CK, (E)EMA, (F)P40, (G)P63, (H) S-100, (I) P53 and (J) Ki-67. (IHC, ×200).

Supplementary Material 2: Fig. 2 IHC staining of recurred tumor in 2015. (A) AE1/AE3, (B)KRT19, (C) KRT7, (D) Pan-CK, (E)EMA, (F)P40, (G)P63, (H) S-100, (I) P53 and (J) Ki-67. (IHC, ×200).

## Data Availability

No datasets were generated or analysed during the current study.
