# Supplementary figures and images for: Loss of clear cell characteristics in aggressive clear cell odontogenic carcinoma: a case report
Source: Diagn Pathol. 2024 Aug 13;19:109. doi: 10.1186/s13000-024-01530-0 (PMC11320854; doi:10.1186/s13000-024-01530-0)

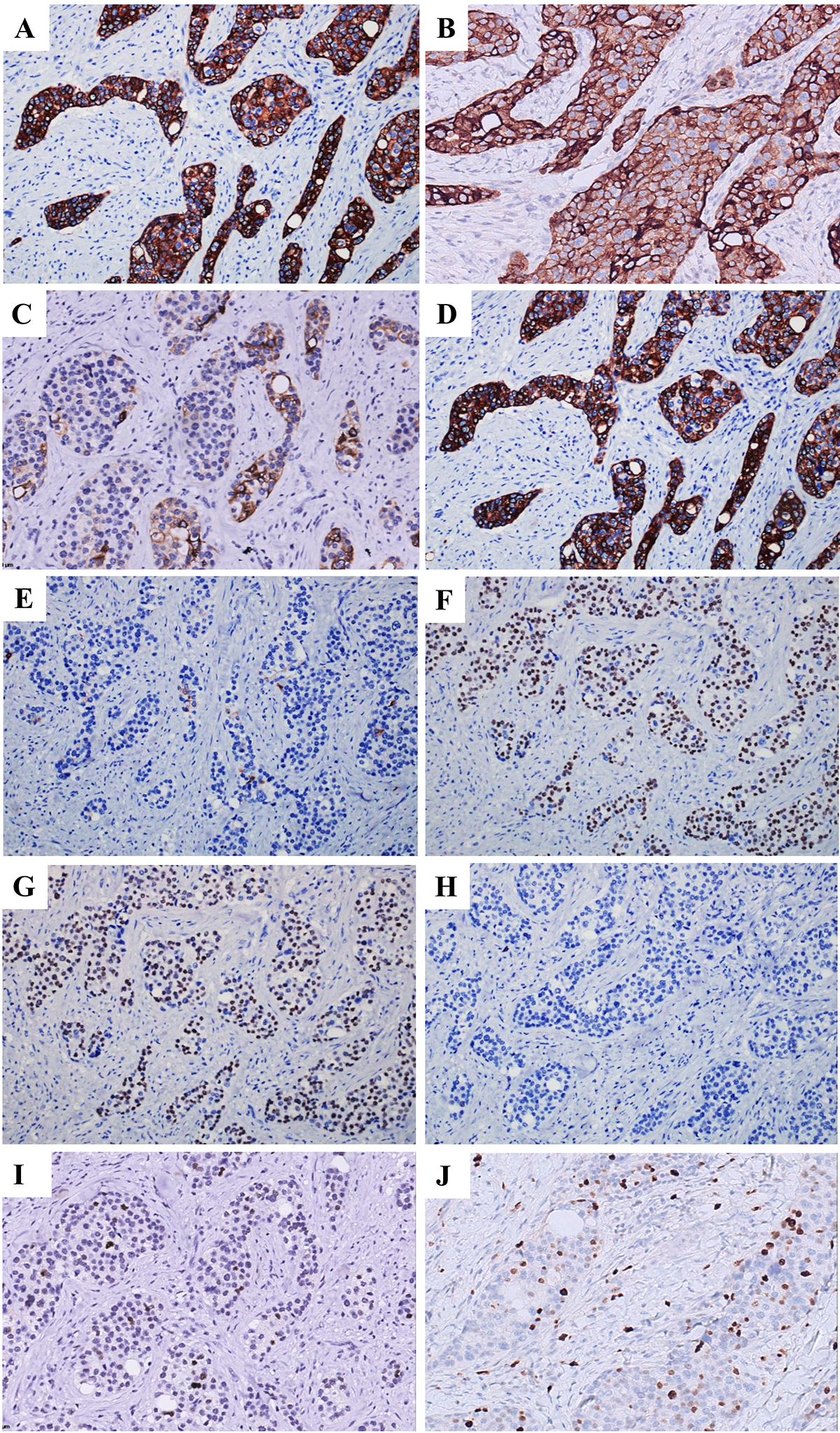

Supplement: Supplementary file 1 — Supplementary Material 1: Fig. 1 IHC staining of tumor in 2012. (A) AE1/AE3, (B)KRT19, (C) KRT7, (D) Pan-CK, (E)EMA, (F)P40, (G)P63, (H) S-100, (I) P53 and (J) Ki-67. (IHC, ×200). [file 13000_2024_1530_MOESM1_ESM.tif]

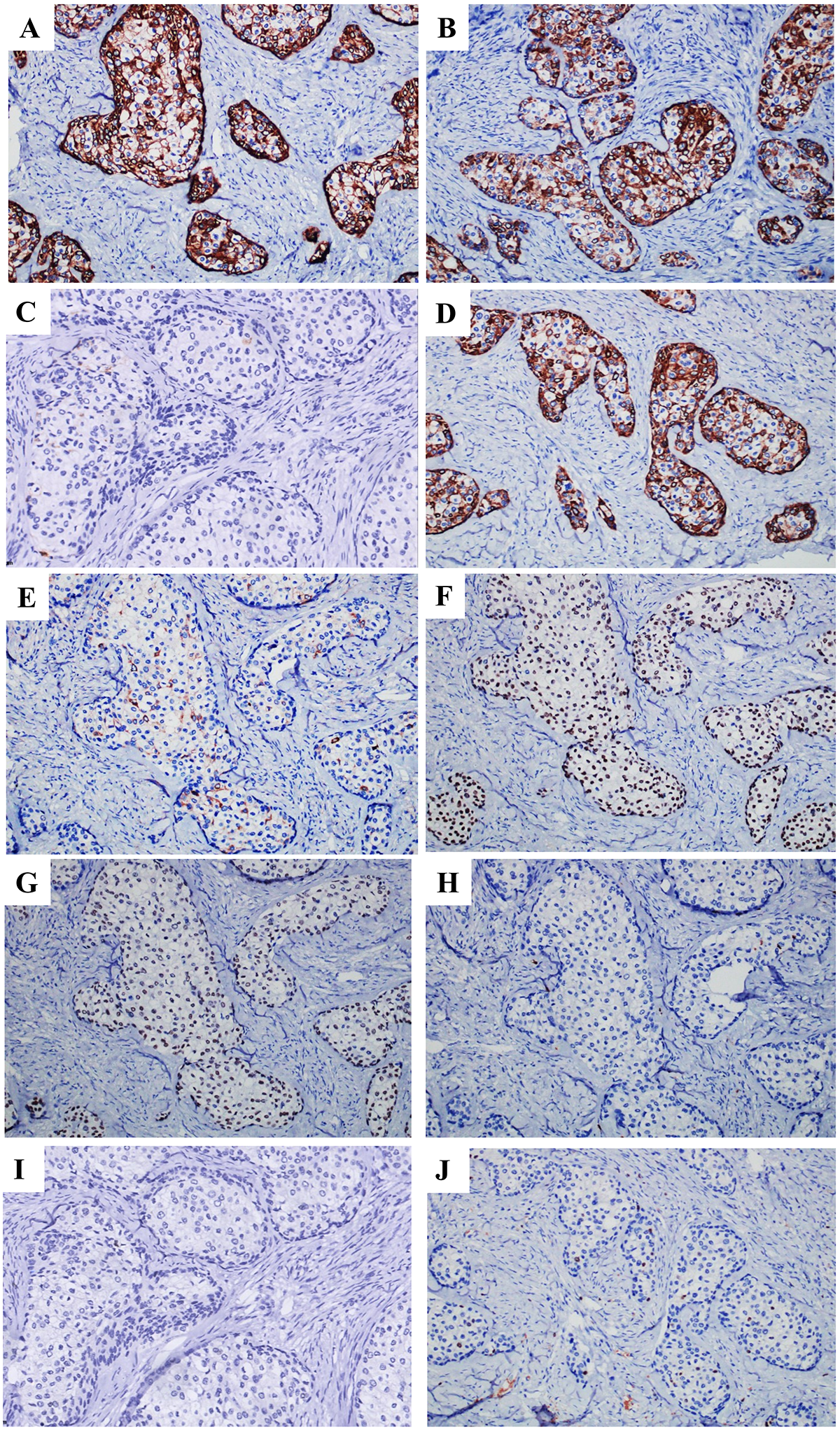

Supplement: Supplementary file 2 — Supplementary Material 2: Fig. 2 IHC staining of recurred tumor in 2015. (A) AE1/AE3, (B)KRT19, (C) KRT7, (D) Pan-CK, (E)EMA, (F)P40, (G)P63, (H) S-100, (I) P53 and (J) Ki-67. (IHC, ×200). [file 13000_2024_1530_MOESM2_ESM.tif]
